# Right Upper Lobe Pulmonary Spindle Cell Neoplasm With Rhabdoid Differentiation and Spinal Canal Invasion in a 19‐Year‐Old Female: A Case Report

**DOI:** 10.1002/ccr3.72743

**Published:** 2026-05-22

**Authors:** Quang Dai La, Aiman Baloch, Sobia Ahmed, Muhammad Ayub, Shanmukh Bachhu, Eric Teng, Hafsa Qayyum, Nam T. Nguyen

**Affiliations:** ^1^ Biology Texas A&M University College Station USA; ^2^ Mekran Medical College Turbat Balochistan Pakistan; ^3^ Department of Radiology Bolan Medical Complex Hospital Quetta Pakistan; ^4^ Civil Engineering, University of California Berkeley Berkeley USA; ^5^ Public Health Texas A&M University College Station USA; ^6^ University of California San Diego USA

**Keywords:** adolescent lung cancer, aggressive thoracic malignancy, multifocal lung tumors, pulmonary spindle cell carcinoma, rhabdoid differentiation, sarcomatoid lung cancer, spinal cord compression, vertebral invasion

## Abstract

We present an unusual case involving a 19‐year‐old female with progressive respiratory distress. Testing revealed pulmonary spindle cell neoplasms with rhabdoid features and spinal cord involvement and infectious complications, emphasizing the importance of multimodal imaging early for rare presentations, histopathological confirmation of diagnosis, and a fully coordinated multidisciplinary team.

## 1 Introduction

Primary pulmonary rhabdoid tumors and spindle cell carcinomas with rhabdoid differentiation are exceptionally rare subtypes of poorly differentiated non‐small cell lung cancers that usually affect middle‐aged adults and confer aggressive clinical behavior [1, 2]. Lung tumors with a rhabdoid phenotype are often sarcomatoid or spindle‐cell tumors, exhibiting both epithelial‐ and mesenchymal‐type morphology. Rhabdoid differentiation is seen across different tissues, and it is a designation that is used in a subset of spindle cell neoplasms from multiple organs. It is characteristically identifiable in histological slides as large polygonal cells with abundant eosinophilic cytoplasm, eccentric nuclei, and frequently a loss of INI1 on immunohistochemistry. Loss of INI1 refers specifically to the loss of expression of the product of the SMARCB1 gene, a tumor suppressor that is also a chromatin remodeling factor, and was predominantly seen in aggressive rhabdoid tumors with high‐grade histologic behavior [1, 3]. Lung tumors with rhabdoid differentiation frequently resemble connective tissue tumors on pathology and have been exhibited in spindle cell carcinoma variants with a terrible prognosis.

Epidemiologically, they account for < 0.4% of all primary lung malignancies, predominantly in male patients aged 56 to 80 years with a median survival rate of three months [4, 5]. Rhabdoid tumors may invade adjacent structures, but can also show aggressive local progression and vertebral and spinal canal invasion if arising from the superior lung fields [3].

The infrequency of primary lung spindle cell tumors in adolescents or young adults, as well as the multifocal disease and spinal invasion, corroborate the clinical and imaging features of our case with previously described cases of pulmonary rhabdoid tumors with spindle cell features. Nevertheless, the literature that addresses multifocal rhabdoid tumors with spinal canal invasion in young patients is extremely limited, and therefore each well‐described case is important for future diagnosis and treatment.

The following case describes a previously healthy 19‐year‐old female presenting with respiratory symptoms due to a large, heterogeneous, cystic‐solid lung mass with vertebral invasion and spinal cord compression, which was later confirmed by histopathology and immunohistochemistry as a spindle cell neoplasm with rhabdoid differentiation. She had stable disease with 6‐month surveillance following chemotherapy, but developed empyema, indicating how challenging this rare thoracic malignancy can be to manage.

This case was presented as a poster at the 40th Annual RSP Radiological Conference in Collaboration with the Royal College of Radiologists (UK) on November 8‐10th, 2024, at the Pearl Continental Hotel, Rawalpindi.

## 2 Case History / Examination

We report a case of a 19‐year‐old female, a resident of a rural periphery, living with her parents and siblings and managing household responsibilities, who was referred to the outpatient department with complaints of a persistent cough for three months, progressive dyspnea for six weeks, and right‐sided chest pain for one month.

There was no history of fever or night sweats and no history suggestive of autoimmune disease or a genetic disorder. There was no family history of lung cancer, and she had never smoked in her life, with no relevant occupational exposure. Multiple empirical courses of antibiotics and painkillers were advised at a rural health center without clinical response.

Her laboratory results showed hypochromia with anisocytosis, an elevated ESR, and mildly elevated CRP. Her sputum Gene Xpert was negative. A chest X‐ray was performed at the rural health center, which demonstrated an inhomogeneous opacity in the right upper lung zone (unfortunately, the image of the CXR is not available).

For further evaluation, she was referred for a contrast‐enhanced CT scan of the chest, which revealed a large heterogeneous mass in the right upper lobe of her lung. The lesion showed internal septations and solid peripheral components. The mass demonstrated direct invasion into the adjacent T3 and T4 vertebrae, with tumor extension into the spinal canal, causing significant spinal cord compression. The mass also displaced the right pulmonary hilum and main bronchus. Interestingly, a similarly appearing mass was identified in the medial basal segment of the left lung, raising concern for multifocal disease.

**FIGURE 1:**
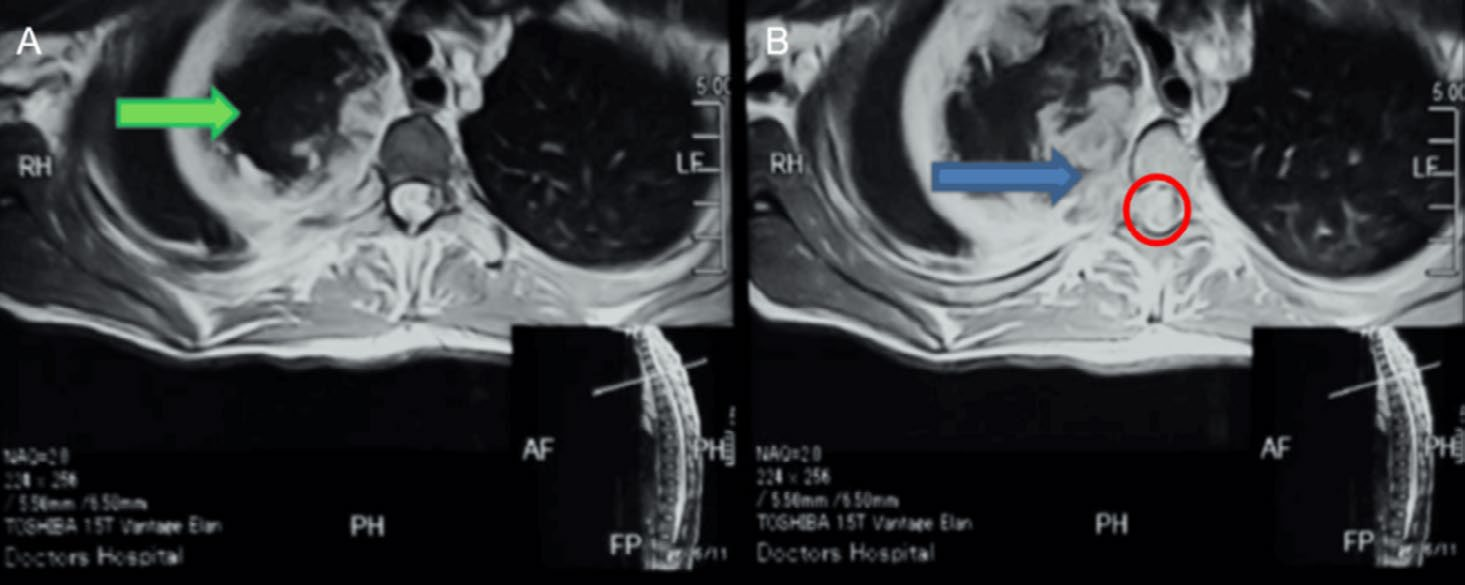
CE MRI showing (A) a large cystic mass with solid nodular peripheral enhancement (green arrow). (B) Spinal canal extension (blue arrow) with compression upon the cord and abnormal signals in the cord. The red circle shows abnormal signs in the cord.

**FIGURE 2:**
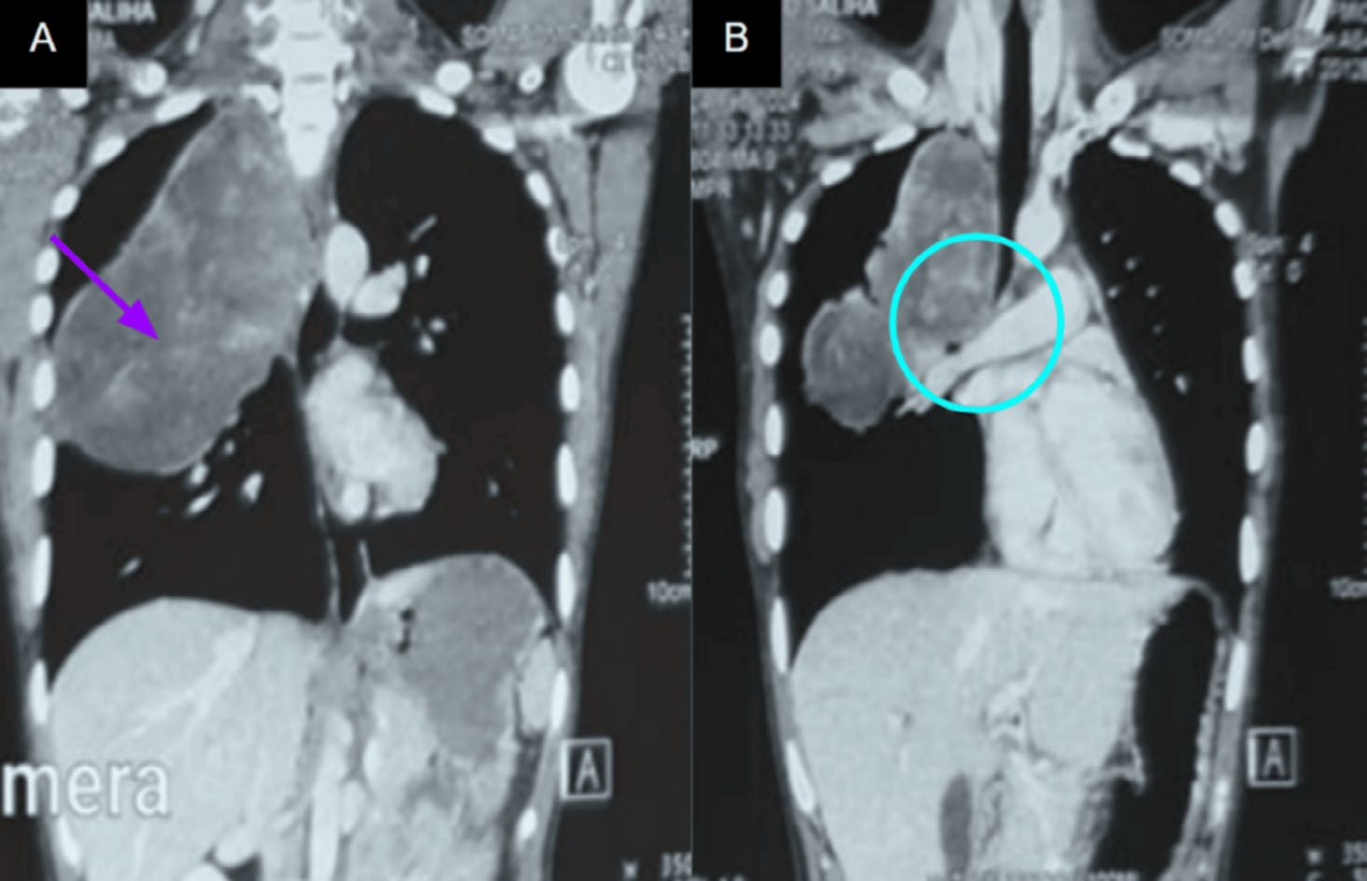
Contrast‐enhanced CT showing the same findings as MRI in Figure 1. (A) A large right upper lobe heterogeneously enhancing mass with internal septations and solid enhancing components predominantly in the periphery of the lesion (purple arrow). (B) It was causing compression upon the right main pulmonary artery and right mainstem bronchus (cyan circle).

**FIGURE 3:**
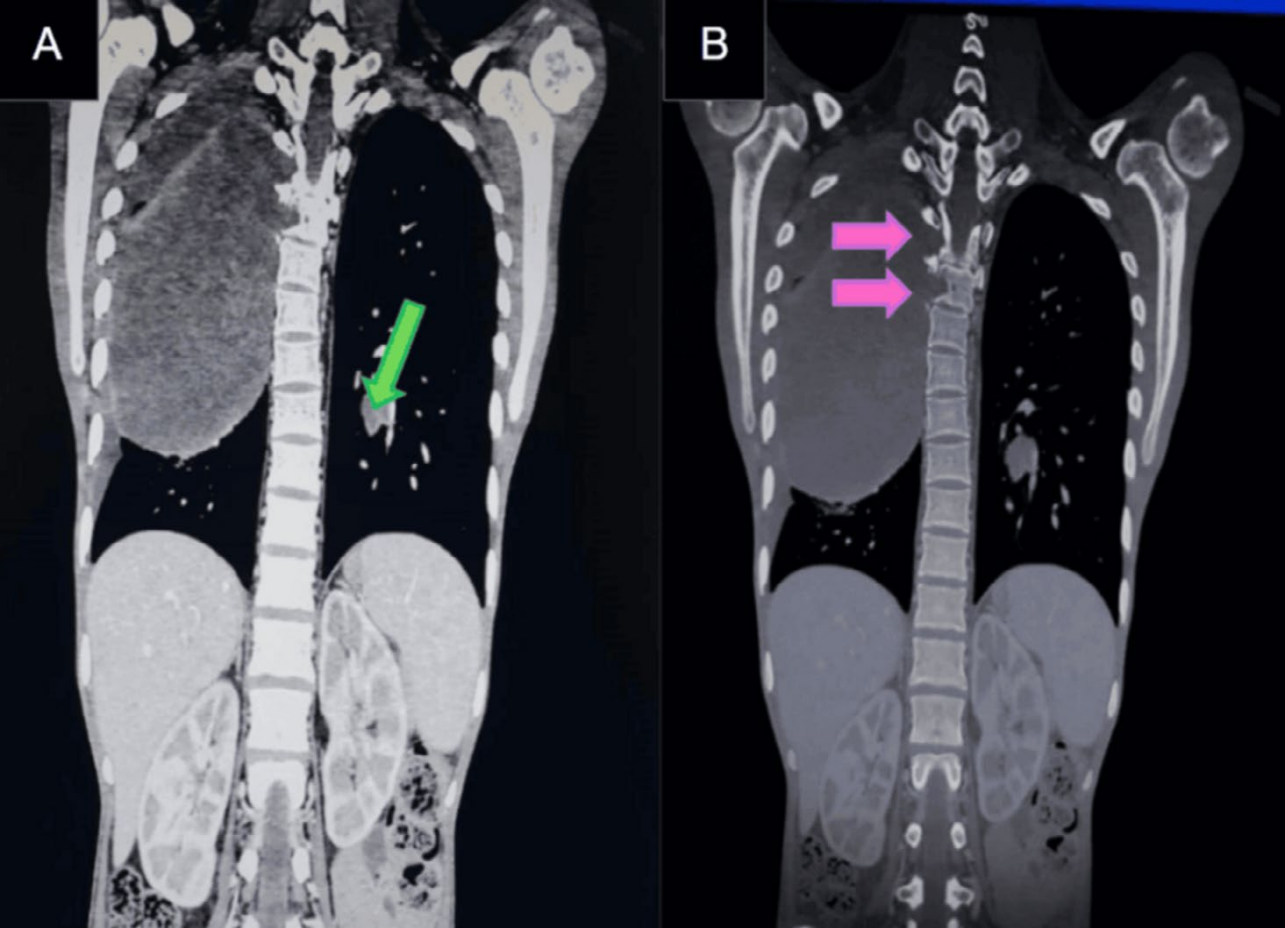
(A) Coronal CT soft tissue window image showing a similar‐looking soft tissue nodule in the contralateral lung (green arrow). (B) Coronal CT bone window image showing vertebral erosions (pink arrows).

Follow‐up thoracic contrast‐enhanced MRIs showed a large cystic mass with solid nodular peripheral enhancement (Figure 1). The mass was still extending into the spinal canal, and there was significant compression of the thoracic spinal cord at T3 as well as a marrow signal abnormality of the involved vertebra (Figure 2). The CT images of the bone and soft tissue windows also showed vertebral erosion, as well as the contralateral pulmonary nodule (Figure 3). These findings support that the aggressive local invasion suggested by the CT and showed the extent of epidural and neural compromise, which are both important for staging and therapeutic planning.

### 2.1 Histopathological and Immunohistochemical Findings

The core needle biopsy sample obtained from the posterior mediastinal mass was initially processed at an outside facility and then reevaluated at a tertiary cancer center. Grossly, the biopsy sample was represented by several gray‐white to dark brown tissue fragments measuring 0.3 × 0.2 × 0.1 cm in aggregate, all submitted for histopathologic analysis, further described in Table 1.

**TABLE 1:** Histopathology and Immunohistochemistry Findings.

Category	Findings
Clinical information	Posterior mediastinal mass; initial differential included lymphoma and neuronal tumor.
Gross description	Multiple gray‐white and dark‐brown tissue fragments measuring 0.3 × 0.2 × 0.1 cm in aggregate. Entirely submitted in one cassette.
Microscopic description	Neoplasm arranged in sheets of spindle to epithelioid cells with abundant eosinophilic cytoplasm, hyperchromatic pleomorphic nuclei, prominent nucleoli, increased mitotic figures, and focal necrosis.
Primary diagnosis	Epithelioid to spindle cell neoplasm.
Recommendation	Immunohistochemical stains are advised for definitive typing. Correlation with clinical and imaging findings is required.
Outside report summary	Similar gross appearance; spindle cell neoplasm with rhabdoid differentiation. Immunophenotyping suggested rhabdomyosarcoma vs. teratoma with rhabdoid features. Diagnosis limited due to biopsy size. Excision advised.

The tumor in these sections of tissue appears to be made up of spindle or epithelioid cells arranged as sheets. The tumor cells were composed of eosinophilic cytoplasm, large undulating nuclei, and large numbers of mitotic figures and necrotic tissue, which are all consistent with high‐grade features. Several other morphologic features were present in the tumor cells, such as rhabdoid appearance with eccentrically located nuclei and ample eosinophilic cytoplasmic material.

The immunohistochemical findings (Table 1) further support our interpretation. Tumor cells exhibited focal positivity for Myogenin and MyoD1 and were positive for Desmin, establishing myogenic differentiation. SOX10, CD68, and SALL4 were negative in tumor cells, thereby ruling out neural crest‐derived tumors, histiocytic lesions, and germ cell tumors, respectively. The lack of SALL4 reactivity strongly suggests against mediastinal germ cell tumor, while SOX10 negativity rules out malignant peripheral nerve sheath tumors. CD68 negativity rules out histiocytic differentiation.

Based on the morphological characteristics of a high‐grade spindle cell malignancy with focal rhabdoid features, in addition to immunophenotypic confirmation of myogenic differentiation and exclusion of other lineages, the diagnosis of spindle cell malignancy with rhabdoid differentiation is made. In the setting of radiographic evidence of a primary pulmonary malignancy with vertebral body invasion, these histopathologic findings are consistent with aggressive pulmonary spindle cell carcinoma with rhabdoid differentiation.

Although an excision was advised due to the limited biopsy sample, the combined clinicopathologic data are already highly suggestive of the final diagnosis.

## 3 Differential Diagnosis

Because of the patient's age and imaging findings, some differential diagnoses included lymphoma and primary neuronal tumors. Given the spindle cell morphology and rhabdoid features, additional considerations included rhabdomyosarcoma and teratoma with rhabdoid differentiation.

## 4 Investigations and Treatment

Histopathologic and immunohistochemical evaluation of the right lung lesion confirmed a spindle cell neoplasm with rhabdoid differentiation (Tables 1 and 2). The patient received several rounds of systemic chemotherapy.

**TABLE 2:** Immuno/Histochemical Stain Results.

Immuno/Histochemical stain (s)	Result
Myogenin	Focal positive
Myo D1	Focal positive
Desmin	Positive
SOX10	Negative in tumor cells
CD68	Negative in tumor cells
SALL4	Negative

*Note:* Myogenin is a transcription factor and skeletal muscle differentiation marker. Myo D1 is a myogenic regulatory transcription factor used as a skeletal muscle lineage marker. Desmin is an intermediate filament protein serving as a broad muscle marker spanning skeletal, smooth, and cardiac subtypes. SOX10 is a transcription factor associated with neural crest, melanocytic, and schwannian lineages. CD68 is a lysosomal glycoprotein marking macrophage and histiocytic lineages. SALL4 is a transcription factor associated with germ cell and primitive/embryonal tumors. “Positive” indicates diffuse or strong immunoreactivity in tumor cells. “Focal positive” indicates immunoreactivity present in only a subset or minority of tumor cells. “Negative” denotes absent immunoreactivity in tumor cells. “Negative in tumor cells” specifies absence within the neoplastic population specifically, as background non‐neoplastic elements such as stromal macrophages or vessels may still stain. The panel supports skeletal muscle/rhabdomyoblastic differentiation given positivity for Myogenin, MyoD1, and Desmin, while negativity for SOX10, CD68, and SALL4 argues against neural crest, histiocytic, and germ cell lineages, respectively.

## 5 Conclusion and Results

**FIGURE 4:**
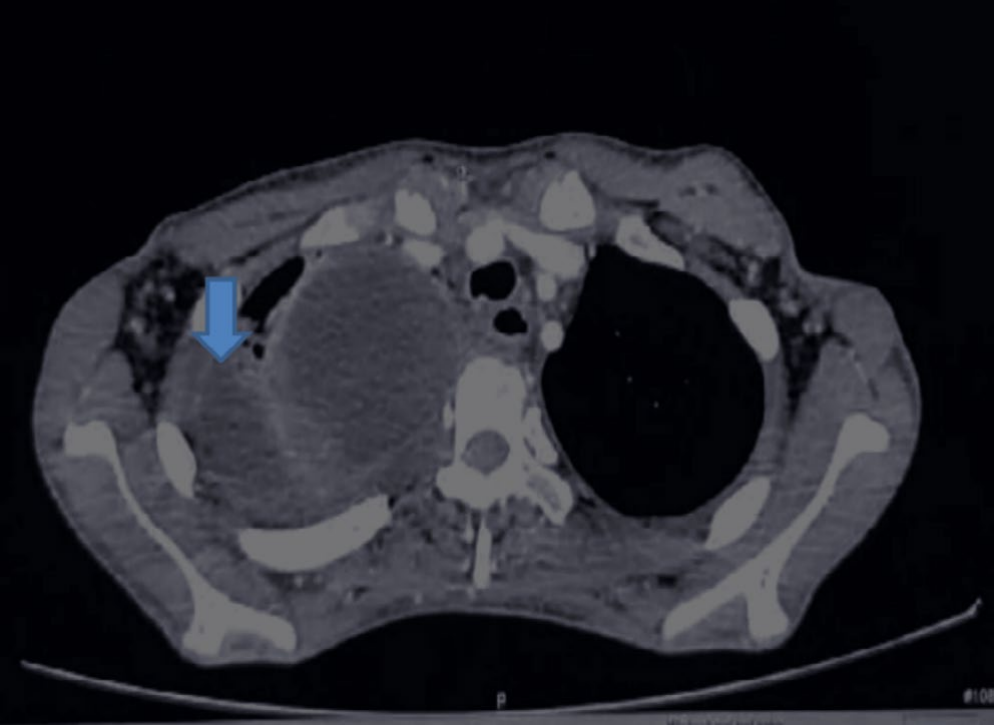
Scan showing empyema (arrow).

A follow‐up CT scan six months later showed stable disease progression with persistent findings. However, there was an additional finding of empyema, consistent with superimposed infection in the context of a partially necrotic mass (Figure 4).

Given the rarity of spindle cell neoplasms with rhabdoid differentiation in adolescents, and the unique presentation of multifocal bilateral pulmonary masses with vertebral involvement and empyema, this case illustrates the importance of maintaining a high index of suspicion. Coordinated multidisciplinary management involving oncology, neurosurgery, and thoracic surgery was essential in managing complex anatomical involvement to achieve the best functional result in this case, while recognizing the aggressive biology of the disease.

## 6 Discussion

Synovial patterns of differentiation, like rhabdoid morphology, are extremely rare in primary pulmonary spindle cell neoplasms, with few reported cases in the literature. Pulmonary spindle cell carcinoma (PSCC), a type of sarcomatoid lung carcinoma, is known to be aggressive, have a poor prognosis, and progress quickly, although spinal canal invasion is very rare [6, 7, 8]. The current case demonstrates the typical aggressive features of PSCC, namely osseous invasion into the T3‐T4 vertebrae and extension into the neural foramina, but we acknowledge the lack of reported cases [6].

Rhabdoid differentiation within spindle cell neoplasms is when you have cells with huge amounts of eosinophilic cytoplasm and eccentric nuclei, and loss of INI1 expression is frequently seen, and is known to add additional biological aggressiveness [1, 9, 10]. Much of the data for rhabdoid tumors is from the CNS and renal sites, and some of these features have been noted in pulmonary tumors with extraordinarily poor outcomes [3, 9, 11, 12, 13, 14].

There are very few reports of spinal or vertebral invasion by rhabdoid or spindle cell lung neoplasms. Most of the reported cases of spinal rhabdoid tumors are cases of primary central nervous system tumors consisting of atypical teratoid/rhabdoid tumors (AT/RT), typically in children [8, 15, 16, 17]. Case reports of extra‐CNS sarcomatoid tumors invading the spine are exceedingly rare; therefore, this case has significance for its thoracic spinal involvement from primarily having a pulmonary origin.

This case is the first of its kind to represent the point of convergence of three extremely rare clinical occurrences: (1) pulmonary spindle cell carcinoma in a teenager, (2) rhabdoid differentiation in a pulmonary spindle cell tumor, and (3) direct invasion of the spinal canal from a sarcomatoid pulmonary malignancy.

PSCC accounts for < 0.4% of all primary pulmonary malignancies and predominantly occurs in older male patients in the sixth to seventh decade of life [6, 7, 8]. Young patient cases are extremely rare in the literature. Recently documented cases in young female patients have involved spindle cell carcinoma without rhabdoid differentiation and without spinal canal invasion. These cases illustrate the age difference but do not involve the morphologic and invasive characteristics present in this case. Therefore, while young patients have been documented, the combination of rhabdoid differentiation and thoracic spinal extension in an adolescent patient has not been specifically described. The direct invasion of the spinal canal by lung tumors is most often reported in superior sulcus tumors in older patients and is not often associated with sarcomatoid differentiation [6, 7, 8]. Few reports specifically mention rhabdoid or spindle cell differentiation of lung cancers with vertebral and neural foraminal invasion. The presence of osseous destruction of T3‐T4 vertebrae with extension into the neural foramina in this case is therefore a very unusual pattern of spread in a teenager.

The spindle cell morphology of pulmonary neoplasms leads to a wide differential diagnosis, which includes inflammatory myofibroblastic tumor, synovial sarcoma, leiomyosarcoma, malignant peripheral nerve sheath tumor, sarcomatoid mesothelioma, and solitary fibrous tumor. The presence of rhabdoid differentiation further widens the differential diagnosis, which now includes rhabdomyosarcoma and true malignant rhabdoid tumor [1, 9, 10]. Thorough immunohistochemistry is thus required to confirm epithelial differentiation and rule out primary sarcomas and/or metastatic disease.

Another important aspect of diagnosis is to differentiate between primary pulmonary and metastatic sarcoma, as the lung is a frequent site of metastasis. This requires correlation with clinical and radiological evaluation to rule out a primary tumor outside the lung. In this particular case, imaging and histopathological evaluation suggested a primary spindle cell tumor of pulmonary origin with rhabdoid differentiation.

The molecular mechanism of pulmonary neoplasms with rhabdoid differentiation is increasingly associated with the dysfunction of the SWI/SNF chromatin remodeling complex, especially the SMARCB1 (INI1) inactivation, which is associated with dedifferentiation and uncontrolled oncogenic transcription. The coexistence of spindle cell morphology and rhabdoid differentiation in this case may represent an aggressive epithelial‐mesenchymal transition (EMT) process, which is mediated by signaling pathways such as TGF‐β, Wnt/β‐catenin, and PI3K/AKT/mTOR, which are associated with increased invasion, survival signaling, and resistance to apoptosis [18, 19]. Moreover, sarcomatoid lung carcinomas are often characterized by high PD‐L1 expression and an immunosuppressive microenvironment, which are associated with EMT and immune evasion. These findings indicate that the combination of EZH2 inhibition in SMARCB1‐deficient tumors and immune checkpoint inhibitors may be biologically rational therapeutic approaches, although these have not been extensively validated.

Imaging is important for assessing disease burden in sarcomatoid tumors of the lung. Computed tomography and magnetic resonance are vital for visualizing tumor morphology, vertebral involvement or erosion, paravertebral mass extension, and cord compression, all of which require relevant information when planning surgery or radiotherapy [6, 7]. In this case, MRI showed cystic and nodular enhancement within the spinal canal, with invasion of the neural foramina, consistent with the pathology that was confirmed on histopathology.

In spite of the aggressive histology, follow‐up CT at six months post‐chemotherapy showed relative stability of the primary masses. However, the development of a right‐sided empyema illustrates the significant risk of secondary infection in large or necrotic tumors. This was consistent with our observations in poorly responsive sarcomatoid tumors, in which the necrosis led to the potential for superimposed infections.

To the best of current literature, this particular case is a uniquely rare combination of pulmonary spindle cell neoplasm with rhabdoid differentiation and thoracic spinal canal invasion in an adolescent. Each of these is individually rare; together, they form a unique clinicopathologic case that further defines the spectrum of known sarcomatoid lung tumors.

Moreover, the fact that it occurred in a 19‐year‐old female, who does not fit the demographic risk profile, further calls into question the current paradigms that suggest PSCC is predominantly a disease of older males. This could raise the question of whether sarcomatoid lung tumors in adolescents and young adults may not represent a biologically distinct subgroup.

### 6.1 Limitations

As a case report, generalizations cannot be made about prognosis or management. There is also a lack of long‐term follow‐up and molecular characterization information in many reported cases of pulmonary spindle cell and rhabdoid tumors, which further emphasizes the need for cumulative reporting to better define the biological behavior and management strategies of these tumors.

## Author Contributions

**Quang Dai La:** data curation, formal analysis, resources, software, visualization, writing – original draft, writing – review and editing. **Aiman Baloch:** data curation, formal analysis, resources, software, visualization, writing – original draft, writing – review and editing. **Sobia Ahmed:** data curation, supervision, validation, writing – original draft, writing – review and editing. **Muhammad Ayub:** data curation, supervision, validation, writing – original draft, writing – review and editing. **Shanmukh Bachhu:** data curation, formal analysis, resources, software, visualization, writing – original draft, writing – review and editing. **Eric Teng:** data curation, formal analysis, resources, software, visualization, writing – original draft, writing – review and editing. **Hafsa Qayyum:** data curation, supervision, validation, writing – original draft, writing – review and editing. **Nam T. Nguyen:** data curation, formal analysis, resources, software, visualization, writing – original draft, writing – review and editing.

## Funding

The authors have nothing to report.

## Ethics Statement

Our institution does not require ethical approval for reporting individual cases or case series.

## Consent

Written informed consent was obtained from the patient for their anonymized information to be published in this article.

## Conflicts of Interest

The authors declare no conflicts of interest.

## Data Availability

The authors have nothing to report.
